# Protocol of a first-in-human clinical trial to evaluate the safety, tolerability, and preliminary efficacy of the bispecific CD276xCD3 antibody CC-3 in patients with colorectal cancer (CoRe_CC-3)

**DOI:** 10.3389/fonc.2024.1351901

**Published:** 2024-02-12

**Authors:** Susanne Jung, Richard F. Schlenk, Christopher Hackenbruch, Sandra S.L. Roldan Pinzon, Michael Bitzer, Martin Pflügler, Juliane S. Walz, Gundram Jung, Jonas S. Heitmann, Helmut R. Salih

**Affiliations:** ^1^ Clinical Collaboration Unit Translational Immunology, German Cancer Consortium (DKTK), Department of Internal Medicine, University Hospital Tübingen, Tübingen, Germany; ^2^ Cluster of Excellence iFIT (EXC2180) ‘Image-Guided and Functionally Instructed Tumor Therapies’, University of Tübingen, Tübingen, Germany; ^3^ Department of Peptide-based Immunotherapy, Institute of Immunology, University of Tübingen and University Hospital Tübingen, Tübingen, Germany; ^4^ NCT Trial Center, National Center for Tumor Diseases (NCT), German Cancer Research Center (DKFZ) and Heidelberg University Hospital, Heidelberg, Germany; ^5^ Department of Internal Medicine V, Heidelberg University Hospital, Heidelberg, Germany; ^6^ Department of Gastroenterology, Gastrointestinal Oncology, Hepatology, Infectiology and Geriatrics, University Hospital Tübingen, Tübingen, Germany

**Keywords:** colorectal cancer, bispecific antibody, CD276, immunotherapy, clinical trial, translational immunology

## Abstract

**Introduction:**

Colorectal cancer (CRC) is the third most common cancer worldwide in men and women. In the metastasized stage, treatment options and prognosis are limited. To address the high medical need of this patient population, we generated a CD276xCD3 bispecific antibody termed CC-3. CD276 is expressed on CRC cells and on tumor vessels, thereby allowing for a “dual” anticancer effect.

**Methods and analysis:**

This first-in-human clinical study is planned as a prospective multicenter trial, enrolling patients with metastatic CRC after three lines of therapy. During the dose-escalation part, initially, an accelerated titration design with single-patient cohorts is employed. Here, each patient will receive a fixed dose level (starting with 50 µg for the first patient); however, between patients, dose level may be increased by up to 100%, depending on the decision of a safety review committee. Upon occurrence of any adverse events (AEs) grade ≥2, dose-limiting toxicity (DLT), or reaching a dose level of ≥800 µg, the escalation will switch to a standard 3 + 3 dose design. After maximum tolerated dose (MTD) has been determined, defined as no more than one of the six patients experiencing DLT, an additional 14 patients receive CC-3 at the MTD level in the dose-expansion phase. Primary endpoints are incidence and severity of AEs, as well as the best objective response to the treatment according to response evaluation criteria in solid tumors (RECIST) 1.1. Secondary endpoints include overall safety, efficacy, survival, quality of life, and pharmacokinetic investigations.

**Ethics and dissemination:**

The CD276xCD3 study was approved by the Ethics Committee of the Medical Faculty of the Heinrich Heine University Düsseldorf and the Paul-Ehrlich-Institut (P00702). Clinical trial results will be published in peer-reviewed journals. Trial registration numbers: ClinicalTrials.cov Registry (NCT05999396) and EU ClinicalTrials Registry (EU trial number 2022-503084-15-00).

## 1 Introduction

Colorectal cancer (CRC) is the third most common cancer entity worldwide in men and women (1). It causes approximately 242,000 deaths per year in Europe (2), mainly due to the dismal 5-year overall survival (OS) rates of approximately 20% or below for metastasized disease (3–5). Particularly for the latter, there is a high medical need for new therapeutic strategies. Present treatment options include combinatorial chemotherapy, kinase inhibitors, and various antibodies (6). However, the latter acts mainly by inhibiting growth factors or angiogenesis, as opposed to directly targeting the tumor cells, with limited benefits only.

Antibodies in different formats have achieved considerable success in cancer therapy. A critical issue for their development is the choice of suitable target molecules, i.e., tumor-associated antigens (TAAs) on cancer cells (7, 8), with their abundance, homogeneity, and stability of expression being of particular importance. Additional expression of a target antigen not only on tumor cells but also on structures critical for tumor growth may yield additional benefit. Such structures that are critical for tumor growth are, for example, extracellular matrix and/or neovasculature. Some TAAs further affect tumor immune surveillance. A prominent example is the programmed death ligand 1/ programmed cell death protein1 (PD-L1/PD-1) signaling pathway, which acts as immune escape mechanism. Blocking this pathway, the so-called immune checkpoint inhibition (ICI) can induce regression even of established tumors, and ICI has become standard treatment in several tumor entities, e.g., melanoma (9) and lung cancer (10). In CRC, however, except for the approximately 15% of patients with microsatellite instability (11, 12), which is a rate that drops to 5% in patients with metastasized disease (13, 14), ICI is not effective (15–18).

Other T-cell recruiting strategies that have recently revolutionized cancer treatment comprise bispecific antibodies (bsAbs), which stimulate the T-cell receptor/CD3 complex with their effector part after binding to their target antigen on tumor cells and the functionally closely related chimeric antigen receptor (CAR) T cells. So far, both bsAbs and CAR T cells are only established in hematologic malignancies but not in solid tumors. This may be largely due to lacking access of immune effector cells to the tumor site (19, 20) and may be overcome by targeting antigens expressed not only by the tumor cells themselves but also by the neovasculature/extracellular matrix. This, in turn, may induce a pro-inflammatory microenvironment, facilitating the influx of effector cells (21, 22).

Efficacy of currently available bsAbs and CAR T cells is additionally hampered by side effects due to unspecific activation of the T cells, resulting in a potentially lethal cytokine release syndrome (CRS) (23, 24). This is due to the employed target antigens frequently being not exclusively expressed not only on malignant but also on healthy cells. Thereby, not only are the patients endangered, but also the application of truly effective doses prevented. Recently, we have developed the PSMAxCD3 bsAb CC-1 in an optimized immunoglobulin G (IgG)–based bsAb format (IgGsc) that displays a prolonged half-life and strictly target-restricted activity with accordingly reduced off-target toxicity (25). CC-1 is currently clinically evaluated in patients with prostate cancer (NCT04104607 and NCT05646550) (26). Recently, the target antigen CD276 (also known as B7-H3) has received large interest due to its broad but rather tumor-restricted expression on cancer cells and neovasculature/microenvironment, including CRC (27, 28). Although this molecule has been shown to have costimulatory effects on T cells in mouse models, it is, meanwhile, considered mainly as inhibitor of human T-cell function, e.g., by suppressing interleukin-2 (IL-2) (27). Preparative work for this clinical trial revealed relevant CD276 expression in all investigated metastatic CRC samples. This prompted us to generate a CD276xCD3 bsAb in our IgGsc format CC-3 that, like CC-1, exhibits an optimal half-life but lacks unspecific T-cell activation while having potent antitumor activity (29) intended for treatment of CRC. Presently widely used targets for CAR T cells and bsAbs in preclinical and early clinical stages of development for CRC treatment are epidermal growth factor receptor (EGFR) and carcinoembryonic antigen (CEA) (19, 30, 31). Compared with these and other immunotherapeutics currently in development, targeting CD276 with CC-3 is expected to

- cause fewer side effects due to its optimized bsAb format and choice of TAA, which will allow for the application of effective bsAb doses and accordingly increased efficacy;
- enable “dual targeting” of tumor cells and neovasculature, which will improve the immune effector cells’ access to CRC tumors, facilitating therapeutic success;
- be a readily available “off the shelf drug,” eliminating the preparatory work required for CAR T-cell production delaying treatment; and
- allow for convenient dosing schedules according to its prolonged half-life (32).

In our first-in-human (FIH) study reported here, we plan to evaluate CC-3 in patients with metastatic CRC to determine the overall safety and tolerability, as well as the MTD and first signs of efficacy.

## 2 Methods and analysis

### 2.1 Properties of bsAb CC-3

The bispecific CD276xCD3 antibody CC-3 is an optimized IgG-like molecule (IgGsc format) with substantially improved serum half-life, especially compared with the prototypical BiTE^®^ bsAb format (29). CC-3 was developed on the basis of extensive preliminary work of our group (33), including the development of the PSMAxCD3 bsAb CC-1 in the same format, which shares with CC-3 the reduced biological risk of side effects (25, 26). *In vitro* and *in vivo* analyses with CC-3 show a favorable toxicity profile, comparable with that of CC-1, of which the target dose has been safely applied in the FIH trial. In addition, *in vivo* data in NSG mice show a highly effective tumor eradication (29).

### 2.2 Study design

This open-label, multicenter, FIH phase I dose-escalation and dose-expansion study is designed to define the MTD and recommended phase II dose (RP2D) of CC-3 for a subsequent phase II study. In addition, the aim of the dose-expansion part is to collect first evidence for efficacy of the MTD of CC-3 in adult patients with CRC after the third-line therapy. The study is entitled “First-in-human clinical trial to evaluate the safety, tolerability, and preliminary efficacy of the bispecific CD276xCD3 antibody CC-3 in patients with colorectal cancer“ (short title: “CoRe_CC-3”) and will be conducted within the framework of the German Cancer Consortium (DKTK).

### 2.3 Study approach

**Figure 1:**
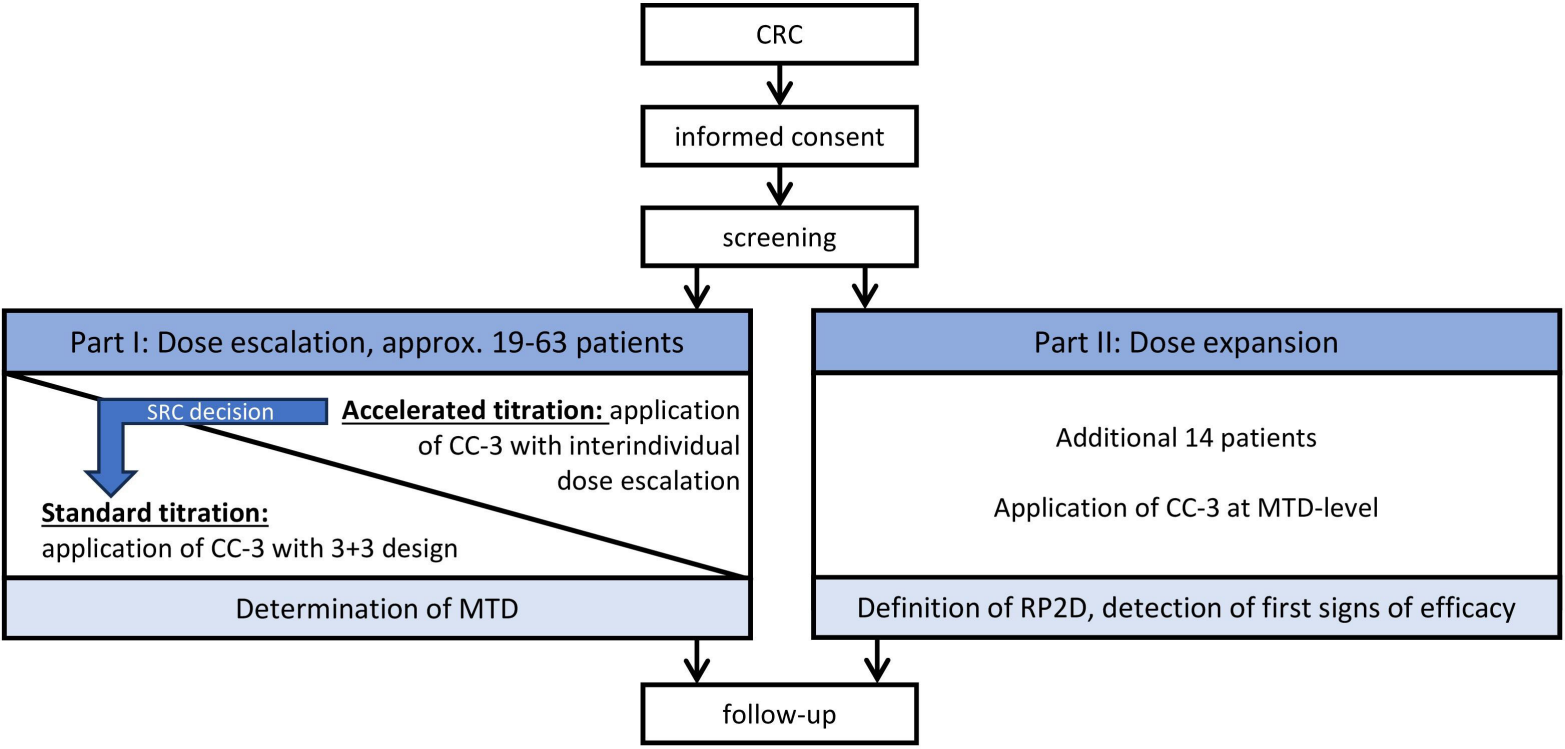
Study overview. CRC, metastasized colorectal carcinoma; CC-3, bispecific CD276xCD3 antibody; SRC, safety review committee; MTD, maximum tolerated dose; RP2D, recommended phase II dose.

**Figure 2:**
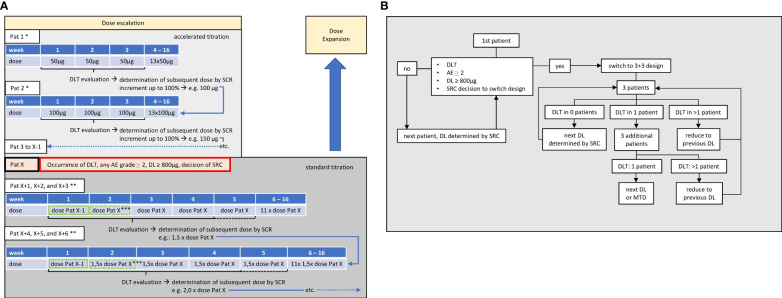
Dose-escalation part. **(A)** Dosing. First patient (Pat 1) receives 50 µg weekly over 16 weeks. During 3 weeks after first application, dose-limiting toxicity (DLT) will be evaluated and the dose level (DL) for the next patient (Pat 2) will be determined by safety review committee (SRC). Repetition of SRC-guided accelerated dose escalation with following patients until occurrence of DLT, AEs ≥2, or decision of SRC, in patient X (Pat X). Switch to 3 + 3 design with last DL before reaching a criterion (Pat X-1) being used as a priming dose (green frame) for the first application, followed by the DL at which the stopping criterion was observed (Pat X) in subsequent applications. When last patient of each cohort has been observed for 21 days after application of target dose, DLT will be evaluated and the dose level for next cohort of at least three patients is determined by SRC. * up to two additional subjects may be enrolled at each DL; ** up to six additional subjects may be enrolled at each DL; *** SRC may decide to add one additional priming dose, so that target dose is reached at week 3 and DLT evaluation period thus prolonged until end of week 4 (dashed lines). **(B)** Flowchart. If no DLT, AEs grade ≥2, or respective decision of SRC occurs, dose levels may be incremented by up to 100% during accelerated dose titration, enabling a rapid increase up to the maximum target dose after a potential minimum of eight patients. After switching to 3 + 3 design, the number of patients per cohort depends on occurrence of DLT, until maximum tolerated dose (MTD) is reached.

This FIH study will consist of two parts: a dose-escalation part comprising an accelerated titration phase in which the dose level (DL) is fixed for the individual patient but may increase with each patient by up to 100%, depending on safety considerations judged by an SRC. In case of CC-3–induced adverse events (AEs) ≥grade 2 according to Common Terminology Criteria for Adverse Events (CTCAE) version 5.0, dose-limiting toxicity, reaching a DL of ≥800 µg or SRC decision, this accelerated titration phase will be switched to a standard 3 + 3 dose–escalation design. At this stage, dosing will be split in a priming dose (last DL of the accelerated titration part with no AE ≥2 and no DLT) and a target dose (DL at which the stopping criterion was observed that terminated the accelerated titration part). Three patients will be enrolled in this first cohort. If one of these three develops DLT, then three additional patients will be recruited into the cohort. If two patients develop DLT, then the dose is de-escalated to the last DL considered as safe. If no patient of this cohort experiences DLT, then the next DL will be determined by the SRC and applied to a second cohort. This will again consist of three patients and follow the same DLT considerations as the previous cohort. This will be repeated until MTD is determined, with MTD defined as no more than one of the six patients experiencing DLT. Once the MTD is determined, dosing will be fixed at this level. Then, further patients will be recruited until 20 patients altogether have received CC-3 at the MTD. This dose-expansion part will serve to confirm the RP2D and to detect first signs of clinical efficacy (see also **Figures 1**, **2**).

### 2.4 Justification of starting and target dose

The definition of the starting dose of 50 µg for CC-3 was based on preclinical data derived from CC-3 dose titrations of *in vitro* T-cell activation and cytokine release, in comparison with pharmacokinetic (PK) data from a clinical study with CC-1 (a bsAb with PSMAxCD3 specificity of otherwise identical format), as well as on clinical experience and clinical studies with MGD009 [a bsAb with CD276xCD3 specificity in a different format (34)] and CD20xCD3 bsAb (35). The maximum dose of 4,000 µg of CC-3 was derived from an *in vivo* model with humanized NOD scid gamma (NSG) mice, in which repetitive dosing with 1.4 µg of CC-3 achieved an eradication of established flank tumors. This dose corresponds to approximately 4,000 µg per week in humans.

### 2.5 Inclusion criteria

Patients must meet the following criteria prior to treatment in the CC-3 study:

- Provision of a written informed consent;
- Patient is able to understand and comply with the protocol for the duration of the clinical trial including undergoing treatment and scheduled visits and examinations;
- Progressive metastatic CRC. Patient must have received three or more prior systemic therapy regimens in the metastatic situation, including FOLFOX, FOLFIRI, FOLFOXIRI, TAS-102, or regorafenib if applicable, in combination with anti–vascular endothelial growth factor (VEGF/VEGFR) monoclonal antibody (mAb) and anti-EGFR mAb in rat sarcoma (RAS) wild-type and left-sided tumors. Exceptions:

  ○ Patients with microsatellite instability (MSI)–high/deficient DNA mismatch repair (MMR) tumors should have received ICI therapy and at least two further therapy regimens of those stated above;
  ○ Patients with V-Raf Murine Sarcoma Viral Oncogene Homolog B (BRAF)V600E mutation should have received cetuximab in combination with encorafenib in second- or third-line treatment; and
  ○ Patients who are considered unsuitable or unwilling to receive further available standard therapy.
At least one measurable lesion that can be accurately assessed at baseline by computed tomography (CT) or magnetic resonance imaging (MRI) and is suitable for repeated assessment per RECIST 1.1;

- Eastern Cooperative Oncology Group (ECOG) performance status ≤2;
- Age ≥18 years, no upper limit;
- Female patients of childbearing potential (FCBP) and male patients with partners of child bearing potential who are sexually active must agree to the use of two effective forms (at least one highly effective method) of contraception. This should be started at signing of informed consent and continue throughout the period in which study treatment is taken and for 2 months after the last dose of CC-3;
- For FCBP, two negative pregnancy tests (sensitivity of at least 25 mlU/mL) prior to first application of CC-3;
- All subjects must agree to refrain from donating blood while on the study drug and for 2 months after the last dose of CC-3;
- Adequate bone marrow, renal, and hepatic function defined by laboratory tests within 14 days prior to clinical treatment:

  ○ Hemoglobin ≥9 g/dL (transfusion of packed red blood cells prior to enrollment allowed);
  ○ Neutrophil count ≥1.500/mm³;
  ○ Platelet count ≥75.000/µL;
  ○ Serum creatinine ≤1.5 mg/dL or creatinine clearance ≥60 mL/min;
  ○ Hepatic function of patients without current hepatic metastasis:
  ◼ Bilirubin ≤1.5 × upper limit of normal (ULN); in case of known Gilbert syndrome, higher values are allowed if due to increase of indirect bilirubin
  ◼ Alanine aminotransferase (ALT) and aspartate aminotransferase (AST) ≤2.5 × ULN.
  ○ Hepatic function of patients with current hepatic metastasis:
  ◼ Bilirubin ≤2.5 × ULN;
  ◼ ALT and AST ≤5 × ULN.

### 2.6 Exclusion criteria

Patients fulfilling any of the following criteria cannot be enrolled in the CC-3 trial:

- Other malignancy requiring treatment within the last year except: adequately treated non-melanoma skin cancer and low-grade non-muscle invasive papillary bladder cancer;
- Concurrent or previous treatment within 30 days before the first CC-3 application in another interventional clinical trial with an investigational anticancer therapy;
- Persistent toxicity (≥grade 2 according to CTCAE version 5.0) caused by previous cancer therapy, excluding alopecia and neurotoxicity;
• Clinical signs of active infection (>grade 2 according to CTCAE version 5.0);
- Known cerebral/meningeal manifestation of CRC;
- History of human immunodeficiency virus infection;
- Active or chronic viral hepatitis (hepatitis B or hepatitis C);
- Ongoing autoimmune disease;
- History of relevant central nervous system (CNS) pathology or current relevant CNS pathology (e.g., seizures, paresis, aphasia, cerebrovascular ischemia/hemorrhage, severe brain injuries, dementia, Parkinson’s disease, cerebellar disease, organic brain syndrome, psychosis, and coordination or movement disorder);
- Therapeutic anticoagulation therapy;
- Major surgery within 4 weeks of starting study treatment. Patients must have recovered from any effects of major surgery;
- Patients receiving any systemic chemotherapy, mAb, or radiotherapy within 2 (for mAb 4) weeks prior to study treatment or a longer period depending on the defined characteristics of the agents used;
- Heart failure New York Heart Association (NYHA) classification III/IV;
- Severe obstructive or restrictive ventilation disorder;
- Intolerance or hypersensitivity to CC-3 (including any excipient present in CC-3) or other immunoglobulin drug products;
- Live and live-attenuated vaccination 30 days prior to treatment;
- Pregnant or breastfeeding women;
- Current ileus with severely altered gastrointestinal function.

### 2.7 Treatment/study distribution

**Figure 3:**
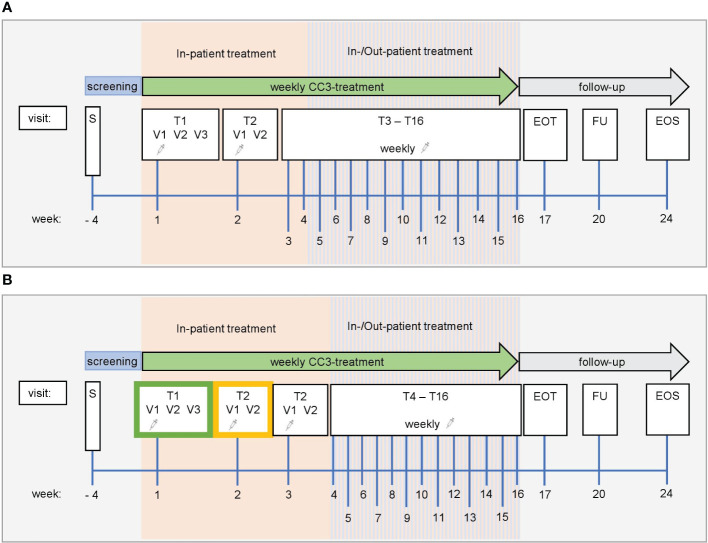
Study schedule. Screening ≤28 days. **(A)** Accelerated titration phase. Dose level in the accelerated titration phase is fixed in each patient and is applied once per week. Dose increase in the next patient can be up to 100% based on safety, PK, and PDK data. For the first application of CC-3 at visit T1V1, patients must be treated as in-patients and followed for safety reasons for at least 48 h Subsequent applications of CC-3 are conducted as in-patient with one overnight stay. If well tolerated and upon medical evaluation, the following doses may be administered in an out-patient setting. In-patient treatment (hospitalization for one night) is feasible and allowed for any subsequent CC-3 treatment. **(B)** Standard titration phase. Dose level at which no AE ≥2 and no DLT was observed during the accelerated titration phase is used as priming dose in the first cohort of enrolled patients (green frame). At T2V1, patients receive DL at which stopping criterion was observed in accelerated titration phase. SRC can decide to introduce an additional second (higher) priming dose (yellow frame) to be applied at T2V1, and dose escalation is conducted at T3V1. For the first application of CC-3 at visit T1V1, patients must be treated as in-patients and followed for safety reasons for at least 48 h Subsequent applications of CC-3 are also conducted as in-patient visits with a single overnight stay. After application of at least three target doses was well tolerated and upon medical evaluation, the following target doses may be administered in an out-patient setting. In-patient treatment (hospitalization for one night) is still feasible and allowed for any subsequent CC-3 treatment. EOT, end of treatment; FU, follow-up; EOS, end of study; S, screening; T, treatment cycle; V, visit.

The study will be divided into two parts. In both, patients receive CC-3 monotherapy as short-term infusion over 3 h once per week, with up to 16 applications per patient if no criterion for treatment termination is met (see treatment schedule in **Figure 3**). Depending on the tolerability of the first three doses, which are always applied in the inpatient setting, further applications may be applied in an outpatient setting at the investigator’s discretion. The treatment phase is followed by a treatment free period of 8 weeks with weekly follow-up visits; the end of safety follow-up visit is thus scheduled for week 24. The duration of the trial for each patient is expected to be 6 months. Thereafter, patients will receive routine treatment by the admitting oncology center. Long-term follow-up data will be collected by telephone calls outside the clinical trial protocol for 3 years. In case of a confirmed benefit of the CC-3 treatment within the clinical trial, a continuation of CC-3 treatment outside the study is possible. A possible continuation will be based on a case-by-case decision by the investigator and the SRC.

#### 2.7.1 Dose-escalation part

Initially, dose escalation will be conducted using an accelerated titration design with single-patient cohorts for each DL. Up to two additional subjects may be enrolled at each DL in case more PK, pharmacodynamic (PDK), or safety data are deemed necessary by the SRC. A staggered enrollment design is used for each DL, with a minimum observation period of 72 h between two consecutive subjects’ first doses with the same DL. This is considered sufficient to identify CRS as most critical toxicity. Every patient receives a fixed dose, starting with 50 µg in the first cohort. Subsequent doses are determined by the SRC based on PK, PDK, and safety data obtained with the last DL over an observation period of 21 days, during which DLT evaluation is conducted. This accelerated phase will be replaced by a standard 3 + 3 design in case of

- any AE grade ≥2 (except AEs unequivocally due to underlying disease or an extraneous cause);
- occurrence of DLT;
- DL ≥800 µg; and
- decision of SRC based on PK, PDK, and safety data.

In the standard titration phase of the dose-escalation part, the last DL safely applied prior to termination of the accelerated phase is used as priming dose upon first application, followed by the DL at which the stopping criterion was observed in week two and thereafter. Based on PK, PDK, and safety data, the SRC may decide to introduce an additional intermittent priming dose at week 2, in which case the target dose will be applied at week 3. At least three patients are treated per cohort, following a rule-based 3 + 3 design. Up to a maximum of nine patients per cohort may be enrolled if more PK, PDK, or safety data are deemed necessary by the SRC. After completion of the first cohort’s DLT evaluation period (21 days after application of the target dose to the last patient), the DL for the next cohort will be determined by the SRC. This will be done considering all AEs and DLTs as well as PK, PDK, and safety data. The DL can be increased up to 100% of the previous dose. The priming dose applied in the first cohort will further be given at week 1 of every consecutive cohort, followed by the target dose at week 2 or 3, depending on whether the SRC deemed an intermittent priming dose necessary at week 2 (**Figure 2A**).

For instance, example 1 (a conservative example) as shown in **Table 1**—(i) a 50% dose increase between cohorts, (ii) one patient treated per cohort in the accelerated titration phase (three cohorts in total), (iii) six patients per cohort in the standard titration phase (10 cohorts in total), (iv) a starting dose of 50 µg, and (v) a maximum tested dose of 4,000 µg—requires a total number of 63 patients in the dose-escalation part.

**Table 1:** Example 1 (a conservative example) of target dose levels in the dose-escalation part.

Level	D1	D2	D3	D3	D4	D5	D6	D7	D8	D9	D10	D11	D12
Cohort	1	2	3	4	5	6	7	8	9	10	11	12	13
Dose (µg)	50	75	113	113*	169	253	380	570	854	1,281	1,922	2,883	4,000
Assumed number of patients per cohort	1	1	1	6	6	6	6	6	6	6	6	6	6
Phase	A	A	A	S	S	S	S	S	S	S	S	S	S

*Standard titration phase is started with new cohorts of initially up to three patients. Treatment schedule is altered to introduce a priming dose on T1V1, leading to application of target dose on T2V1 and consecutive treatment das; A, accelerated titration phase (light gray); S, standard titration phase (dark gray).

Example 2 (a progressive example) as shown in **Table 2**—(i) a 100% dose increase between cohorts, (ii) one patient treated per cohort in the accelerated titration phase (four cohorts in total), (iii) upon a DL of ≥ 800 µg of three additional cohorts with three patients each, (iv) six patients treated in the last cohort of the standard titration phase, (v) a starting dose of 50 µg, and (vi) a maximum tested dose of 4000 µg—requires a total number of 19 patients in the dose-escalation part.

**Table 2:** Example 2 (a progressive example) of target dose levels in the dose-escalation part.

Level	D1	D2	D3	D4	D5	D6	D7	D8
Cohort	1	2	3	4	5	6	7	8
Dose (µg)	50	100	200	400	800	1,600	3,200	4,000
Assumed number of patients per cohort	1	1	1	1	3	3	3	6
Phase	A	A	A	A	S	S	S	S

A, accelerated titration phase (light gray); S, standard titration phase (dark gray).

In case AE-fulfilling criteria for DLT occur, additional patients are enrolled into the cohort. If two patients experience DLT, then the dose is de-escalated to the previous lower DL that was formerly considered to be safe, and, further, patients receive this lower DL until a total of six patients have been treated or further DLTs develop. If two of the four or two of the six patients experience a DLT, then the DL will be de-escalated to the next lower DL formerly considered to be safe, and patients will receive this lower DL until a total of six patients have been treated (**Figure 2B**). MTD is defined as the DL at which none or one patient experienced a DLT. Once the MTD has been defined, the dose-escalation part of the study is terminated and the dose-expansion part commences.

#### 2.7.2 Dose-expansion part

After the dose-escalation part has been terminated, additional patients are treated until a total of 20 patients have received the DL defined as the MTD. During the dose-expansion part, patients can be treated simultaneously. Prior to the start of the dose-expansion part, a substantial amendment including the applied DLs will be submitted to the regulatory authorities.

### 2.8 Endpoints of the study

The primary endpoints of the dose-escalation part are incidence and severity of AEs, including AESIs, serious AEs (SAEs) and suspected unexpected serious adverse reaction (SUSARs) [CTCAE v5.0 and for CRS modified criteria by Lee et al. (36)], from first application of CC-3 at week 1 until week 4 or, in case of early termination, last assessment. The primary endpoints of the dose-escalation and dose-expansion parts are the best objective response, defined as stable disease (SD), partial remission (PR), or complete remission (CR) until end of study (EOS) compared to baseline based on routine imaging according to RECIST 1.1.

Secondary endpoints include general long-term safety, product-specific safety aspects, clinical efficacy endpoints, and pharmacokinetics:

• Safety (general): incidence and severity of AEs including AEs of special interest (AESIs), SAEs, and SUSARs according to CTCAE v5.0 from first application of CC-3 until EOS visit or, in case of early termination, last assessment
• Efficacy:
  ○ Objective tumor response assessed by RECIST 1.1 on routine imaging at week 8, week 16, and at EOS visit;
  ○ Disease control rate (CR, PR, and SD) at each visit until end of the clinical trial.
• Survival:
  ○ OS defined as the time from first administration of CC-3 to time of death from any cause. Patients without the event are censored on the last date of follow-up;
  ○ Progression-free survival defined as the time from first administration of CC-3 to progression of disease or death from any cause, whichever occurs first. Patients without the event are censored on the last date of follow-up.
• Pharmacokinetics: CC-3 serum concentrations assessed at week 1, week 2, and, in case of second priming dose, at week 3
• Quality of life: overall quality-of-life scores (EORTC QLQ C-30) at screening visit, week 5, week 9, week 13, end of treatment (EOT) visit, follow-up (FU) visit, and EOS visit.

Exploratory endpoints include an analysis of PDK and cytokine levels in serum, changes from baseline in the tumor-markers CEA and CA 19-9, as well as a correlation of the effect of CC-3 on serum cytokine levels and PDK biomarkers and a correlation of safety and efficacy of CC-3 treatment with clinical, biological, and patient characteristics. An analysis of CD276 expression is not deemed necessary at the time of enrolment, as an expression of CD276 was shown on all CRC tumor samples analyzed in preliminary assessments. However, if tumor samples are available, then CD276 expression will be evaluated. The rate of patients undergoing secondary resection of liver metastases after initially irresectable disease until EOS will also be assessed.

### 2.9 Safety/dose-limiting toxicity

Toxicity will be graded according to the National Cancer Institute CTCAE (v5.0) and the CRS grading system by Lee et al. (36). All AEs and SAEs will be documented and reported according to good clinical practice guidelines. Furthermore, we will report on AESIs, which include occurrence of CRS, infusion-related reactions upon study drug administration and drug induced liver injury (DILI). These AESIs will be monitored closely and included in patient safety narrative reports to the data safety monitoring board (DSMB).

DLT is defined as any AE grade ≥3 that the investigator considers to be at least possibly related to study treatment and occurring within the DLT safety period (from first study drug treatment to day 21 or 28, respectively, if an additional intermittent priming dose is given) with few exceptions. Notably, grade ≥3 CRS lasting less than 48 h, grade ≥3 nausea, vomiting or diarrhea in patients who have not received optimal antiemetic or antidiarrheic treatment, as well as isolated ≥3 laboratory abnormalities resolving within 7 days and without clinical sequelae are not considered a DLT. Likewise, liver toxicity caused by obstruction, metastasis, or infection; grade ≥3 transaminitis or direct hyperbilirubinemia for <4 days; grade 3 fatigue for ≤5 days; grade 3 infusion reaction resolving within 8 h; nor grade 3 but adjustable (i.e., not refractory) arterial hypertension are not considered a DLT.

### 2.10 SRC and DSMB

Trial conduct will be overseen by an SRC composed of at least three members, including the coordinating investigator and a representative of the sponsor. It receives reports routinely 21 days after first dose of CC-3 of each single patient in the accelerated titration phase and as soon as three patients per cohort have completed the DLT evaluation period of 21 days after target dose application in the standard titration phase. The SRC assesses all available data on safety, PK, and PDK and determines the dose for the next cohort.

The DSMB is composed of three independent experts assessing study progress and safety data. It receives reports routinely after the end of the accelerated titration phase, after the end of the standard titration phase, upon request by the SRC, or in case of occurrence of DLT.

### 2.11 Sample size calculation

The total sample size of the trial is assumed to be approximately 77 patients but depends on the observed safety profile, the number of dose cohorts evaluated, and the number of patients per cohort in the dose-escalation part and can therefore vary. Assuming a dose increase of 50% between cohorts, one treated patient per cohort in the accelerated phase (in total three cohorts), six treated patients per cohort (in total 10 cohorts) in the standard titration phase, a starting dose of 50 µg and a tested dose of 4,000 µg, and the estimated number of patients in the dose-escalation part is 63. In the dose-expansion part, up to 20 patients will be treated with the MTD. In this way, we will be able to estimate within a single-stage phase II design an objective response. For this purpose, objective response will be defined as SD, PR, or CR estimating P0 as the maximum response proportion of a poor drug of ≤20% of the patients and P1 as the minimum response proportion of a good drug of ≥50% with a power of 80% and a type one error of 5%. To this aim, we will need n = 17 evaluable patients assuming a dropout rate of 15% (n = 3).

### 2.12 Patient and public involvement

CC-3 production (in accordance to good manufacturing practice) and trial conduct are exclusively funded by public resources without contribution from pharmaceutical industry or commercial organizations. Prior to approval of funding, proposals were peer reviewed in a competitive manner by the Helmholtz Validation Fund and the Joint Funding Program of the German Cancer Consortium. Extensive interaction consistently maintained with multiple patient representative groups in the framework of the alliance for patient participation in cancer research of the National Center for Tumor diseases (NCT) will substantially support our comprehensive efforts to inform patients about the beginning of study as well as patient recruitment.

### 2.13 Data handling and storage

All findings including clinical, radiological, and laboratory data will be documented by the investigator or an authorized member of the study team in the electronic case report form. The investigators guarantee the privacy of patients. All personal data are treated according to the European general data protection regulation (EU 2016/679) and the German law. The data will be stored for 25 years. All data entry, modification or deletion, will be recorded automatically in an electronic trail. Monitoring of data will be conducted on a regular basis as well as prior to each safety report for SRC and DSMB.

## 3 Discussion

As of now, metastatic CRC is an often rapidly progressive disease without curative treatment options. Here, we introduce a planned FIH study evaluating the safety and efficacy of the bispecific CD276xCD3 antibody CC-3 in patients with CRC after failure of third-line therapy. CC-3 was developed in our laboratory in a novel IgG-like format (IgGsc) to overcome several problems of so far available bsAbs. CC-3 not only comes with a prolonged serum half-life but also acts in a fully target-restricted manner with accordingly reduced off-target T-cell activation, resulting in reduced side effects (37, 38).

CD276, the target antigen of CC-3, is expressed on both tumor cells and tumor vessels of CRC. Thereby, a “dual mode of anticancer action” is enabled: On the one hand, targeting the tumor vessels should allow for improved influx of T cells into the tumor site via the damaged endothelial barrier. This should then be followed by an effective destruction of the tumor cells. Thereby, we expect to overcome a critical factor that so far limits the success of T cell-based immunotherapy of solid tumors (21, 39, 40).

Considering the lack of effective treatment options and the dismal prognosis of the study patient population, the expected benefits of CC-3 treatment in this clinical study outweigh the potential risks, especially because multiple risk mitigation measures have been implemented. The progress and safety data will be closely monitored by an SRC. Moreover, it will be regularly analyzed by the three independent experts of the DSMB. The SRC will carefully supervise the determination of the MTD, ensuring safe treatment of the study patients during dose escalation. The DSMB will counsel the coordinating investigator and the sponsor on whether to alter the trial protocol or discontinue the trial altogether.

In addition, the implemented one-patient-cohort escalation of CC-3 in the accelerated titration part is considered to benefit the patients included in this trial. Usually, the focus of phase I clinical trials is primarily set on safety and tolerability, and patients enrolled during early phases of a trial frequently do not receive sufficiently effective doses. This problem is addressed in our trial by our approach to rapidly increase CC-3 DLs in order to reach preclinically effective doses already in patients included early in the study. If no DLT is observed, then DL may be increased by up to 100% from one patient to the next, enabling an exponential dosage increment allowing to reach the maximum dose of 4,000 µg already with the 14th patient in case no DLT occur. A dose expansion part will then follow the dose-escalation part to collect first signs of efficacy and define the RP2D.

With the CC-3 antibody showing convincing preclinical activity, we expect that the study will demonstrate a corresponding clinical efficacy regarding objective tumor response and disease control rate, leading to a due benefit for this particular patient population through a trial that is fully funded by public resources.

## 4 Ethics and dissemination

The study will be performed in accordance with the Declaration of Helsinki and will comply with the International Conference on Harmonization and Good Clinical Practice. In particular, no study examinations or treatments will be undertaken without first obtaining written informed consent from the patients. This trial is funded by research grants from the Helmholtz Validation Fund (Colomab) and the Joint Funding Program of the DKTK. The CC-3 study was approved by the Ethics Committee of the Medical Faculty of the Heinrich Heine University Düsseldorf and the Paul-Ehrlich-Institut, Germany (P00702). During trial conduct, the responsible authorities will be informed on a regular basis about the progress of the trial. The results of this clinical trial will be presented at relevant national and international meetings and published in peer-reviewed journals regardless of outcome. All planned publications will be reviewed by the principal investigator and the biostatistician prior to publication to avoid violation of patients’ rights.

## Ethics statement

The study will be performed in accordance with the Declaration of Helsinki and will comply with the International Conference on Harmonization and Good Clinical Practice. The CC-3 study was approved by the Ethics Committee of the Medical Faculty of the Heinrich Heine University Düsseldorf and the Paul-Ehrlich-Institut, Germany (P00702).

## Author contributions

SJ: Writing – original draft. RS: Writing – review & editing. CH: Writing – review & editing. SR: Writing – review & editing. MB: Writing – review & editing. MP: Writing – review & editing. JW: Writing – review & editing. GJ: Writing – review & editing. JH: Writing – original draft. HS: Writing – original draft.
